# TATA box-binding protein-related factor 3 drives the mesendoderm specification of human embryonic stem cells by globally interacting with the TATA box of key mesendodermal genes

**DOI:** 10.1186/s13287-020-01711-w

**Published:** 2020-05-24

**Authors:** He Liang, Peng Zhang, Hua-Jun Bai, Jijun Huang, Huang-Tian Yang

**Affiliations:** CAS Key Laboratory of Tissue Microenvironment and Tumor, Laboratory of Molecular Cardiology, Shanghai Institute of Nutrition and Health, University of Chinese Academy of Sciences (CAS), CAS, 320 Yue Yang Rd, Biological Research Building A, Shanghai, 200031 People’s Republic of China

**Keywords:** Human embryonic stem cells (hESCs), TATA box-binding protein-related factor 3 (TRF3), Mesendodermal differentiation, *BRACHYURY* (*T*), *EOMESODERMIN* (*EOMES*), Mix paired-like homeobox (*MIXL1*), GOOSECOID homeobox (*GSC*)

## Abstract

**Background:**

Mesendodermal formation during early gastrulation requires the expression of lineage-specific genes, while the regulatory mechanisms during this process have not yet been fully illustrated. TATA box-binding protein (TBP) and TBP-like factors are general transcription factors responsible for the transcription initiation by recruiting the preinitiation complex to promoter regions. However, the role of TBP family members in the regulation of mesendodermal specification remains largely unknown.

**Methods:**

We used an in vitro mesendodermal differentiation system of human embryonic stem cells (hESCs), combining with the microarray and quantitative polymerase chain reaction (qRT-PCR) analysis, loss of function and gain of function to determine the function of the TBP family member TBP-related factor 3 (TRF3) during mesendodermal differentiation of hESCs. The chromatin immunoprecipitation (ChIP) and biochemistry analysis were used to determine the binding of TRF3 to the promoter region of key mesendodermal genes.

**Results:**

The mesendodermal differentiation of hESCs was confirmed by the microarray gene expression profile, qRT-PCR, and immunocytochemical staining. The expression of TRF3 mRNA was enhanced during mesendodermal differentiation of hESCs. The TRF3 deficiency did not affect the pluripotent marker expression, alkaline phosphatase activity, and cell cycle distribution of undifferentiated hESCs or the expression of early neuroectodermal genes during neuroectodermal differentiation. During the mesendodermal differentiation, the expression of pluripotency markers decreased in both wild-type and TRF3 knockout (TRF3^−/−^) cells, while the TRF3 deficiency crippled the expression of the mesendodermal markers. The reintroduction of TRF3 into the TRF3^−/−^ hESCs rescued inhibited mesendodermal differentiation. Mechanistically, the TRF3 binding profile was significantly shifted to the mesendodermal specification during mesendodermal differentiation of hESCs based on the ChIP-seq data. Moreover, ChIP and ChIP-qPCR analysis showed that TRF3 was enriched at core promoter regions of mesendodermal developmental genes, *EOMESODERMIN*, *BRACHYURY*, mix paired-like homeobox, and GOOSECOID homeobox, during mesendodermal differentiation of hESCs.

**Conclusions:**

These results reveal that the TBP family member TRF3 is dispensable in the undifferentiated hESCs and the early neuroectodermal differentiation. However, it directs mesendodermal lineage commitment of hESCs via specifically promoting the transcription of key mesendodermal transcription factors. These findings provide new insights into the function and mechanisms of the TBP family member in hESC early lineage specification.

## Background

The human embryonic stem cells (hESCs), with the ability of self-renewal and differentiation to derivatives of three germ layers, are a unique model to study human early development [9, 38, 58]. The differentiation of hESCs into mesoderm and endoderm undergoes an intermediate state called mesendoderm (ME), which is equivalent to the primitive streak during gastrulation [55]. During this process, numerous genes, encoding the transcription factors critical for the ME specification, such as EOMESODERMIN (EOMES), BRACHYURY (T), mix paired-like homeobox (MIXL1), and GOOSECOID homeobox (GSC), are transcribed [8, 14, 18, 34]. The fine-tuned spatiotemporal transcription requires the coordination of various signaling pathways, epigenetic modifications, specific transcriptional factors, and general transcription factors (GTFs). Epigenetic modifiers, such as SETD7 and EZH2, are orchestrated to turn on the transcription of ME genes by the induction of wingless-type MMTV integration site family (WNT) and NODAL signals [38, 61]. More recently, we found a key role of SMYD2 in the regulation of ME specification from hESCs via the histone methylation of ME genes [4]. However, the roles of GTFs during the ME differentiation of hESCs remain largely unknown. Deciphering the function of GTFs during early differentiation would not only help us to understand more about the lineage fate commitment, but also facilitate the application of hPSC derivatives in cell therapy and drug development [16, 47, 68].

In eukaryotes, the transcription initiation is a key step in the control of gene expression, which requires the assistance of a large number of GTFs to form a preinitiation complex (PIC) [30]. Typically, the PIC formation starts from the core promoter recognition by TBP/TBP-related factors, which helps to recruit other transcription factors to the promoter regions to initiate the gene expression. To date, four proteins homologous to TBP have been discovered: TBP-related factor 1 (TRF1) and TRF4, described only in *Drosophila melanogaster* [7]; TRF2, broadly existing in many species, including *Drosophila* [50], *Caenorhabditis elegans* [17, 35], *Xenopus* [46], zebrafish [44], chick [42], mouse [66], and human [48, 56]; and TRF3, as known as TBP2 or TBPL2 (TBP like 2), expressed in most vertebrates, from fish to human [49]. TRF3 has been shown to regulate embryogenesis of *Xenopus* [1, 33] and zebrafish [6, 28, 29]. In mouse ESCs, TRF3 and TBP are selectively recruited to different gene promoters [63]. TRF3 is also detected in multiple organs of human, such as the heart, lung, and liver [49]. However, the function and mechanism of TRF3 in human development remain unknown.

In the present study, using the in vitro ME differentiation model of hESCs, combining with the TRF3 knockout (TRF3^−/−^) and rescue, and molecular analysis approaches, we reported a previously unrecognized role of the vertebrate-specific general transcription factor TRF3 and its global regulatory function in the human ME commitment. Our findings provide new insights into the specific role of the TBP family member during the hESC early lineage commitment and uncover the novel mechanism that “GTFs” can switch the hESC state to the early specific lineage by shifting its binding profile.

## Methods

### hESC culture and in vitro differentiation

hESC H1 line (WiCell Research Institute, Madison, WI, USA) was cultured in mTeSR1 media (Stem Cell Technologies, Vancouver, Canada) on Matrigel (Corning, New York, NY, USA) coated dishes as previously reported [4, 12, 31, 32]. To induce the ME and neuroectodermal specification, the media were changed from mTeSR1 to chemically defined medium (CDM) as previously described [4, 11] when hESCs reached approximately 70 to 80% confluency. For the ME induction, hESCs were cultivated in CDM supplemented with Activin A 100 ng/ml (R&D Systems, Minneapolis, USA), bone morphogenetic protein 4 (BMP4) 10 ng/ml (R&D Systems, Minneapolis, USA), basic fibroblast growth factor (bFGF) 20 ng/ml (Invitrogen, Carlsbad, CA, USA), and LY294002 (phosphoinositide-3-kinase/akt serine/threonine kinase inhibitor) 10 μM (Sigma-Aldrich, Carlsbad, USA) for 3 days as described [4, 15]. For the neuroectodermal induction, hESCs were cultivated in CDM supplemented with SB431542 10 μM, a transforming growth factor β receptor inhibitor (Merck, Darmstadt, Germany), and bFGF 12 ng/ml for 7 days as previously described [4, 15].

### Generation of TRF3^−/−^ hESCs and detection of mutation

TRF3^−/−^ hESCs were generated using CRISPR/Cas9 (CRISPR, clustered regularly interspaced short palindromic repeats/Cas9, CRISPR associated 9) technology. The gRNA containing the sequence 5′-ACGTGCTCACGGTCAACGAG-3′ targeting the first exon of TRF3 genome, which was generated with an online tool kit “CHOPCHOP” (http://chopchop.cbu.uib.no/), was constructed into a target vector pSpCas9(BB)-2A-Puro (PX459) (Plasmid #48139, Addgene) [52] and named as PX459-gRNA-TRF3. Then, the constructed PX459-gRNA-TRF3 was nucleofected into H1 hESCs (TRF3^+/+^). Forty-eight hours after nucleofection, puromycin (1 μg/ml) was supplemented to screen the cells containing PX459-gRNA-TRF3 vector. Once the cells amplified into 70–80% confluency, the hESCs were digested into single cells with Accutase (Stem Cell Technologies, Vancouver, Canada) and 1000 cells were re-plated into a 10-cm dish, supplemented with 5 μM rho-associated coiled-coil kinase (ROCK) inhibitor Y-27632 (Merck, Germany). About 2 weeks later, the single cell was propagated into a clone, which is called single cell-derived clone (single clone). Single clones were picked, and two TRF3^−/−^ clones of hESCs (TRF3^−/−^-1 and TRF3^−/−^-2) were confirmed by Sanger sequencing using primers listed in Additional file 1: Table S1.

The off-target site was predicted online with an online tool kit “CHOPCHOP” (http://chopchop.cbu.uib.no). The primers for Sanger sequencing were listed in Additional file 1: Table S1.

### Reintroduction of TRF3 into TRF3^−/−^ hESCs

The human TRF3 cDNA (RC211988, NM_199047, OriGene, USA) was cloned into pCDH-EF1-3×Flag-MCS-T2A-Puro, modified from pCDH-EF1-MCS-T2A-Puro (System Biosciences, CA, USA), and named as pCDH-EF1-3×Flag-TRF3-T2A-Puro. The plasmid was verified by Sanger sequencing, and the vector was served as negative control. The viral package was performed with HEK-293FT cells (cat no. R70007, Invitrogen, Carlsbad, CA, USA) after transfection of plasmids (pMDLg/pRRE, pRSV-Rev, pMD2.G, pCDH plasmid) with lipofectamine 2000 (Invitrogen, Carlsbad, CA, USA) following the manufacturer’s instructions. For the viral infection, TRF3^−/−^-1 and TRF3^−/−^-2 hESCs were infected with the lentiviruses (LV-vector and LV-CDH-EF1-TRF3-T2A-Puro, respectively) for 6 h, and then, the media were changed. Forty-eight hours post-transfection, infected cells were selected with puromycin (1 μg/ml) for 2 days to generate the TRF3 overexpression cell lines with TRF3^−/−^-1 and TRF3^−/−^-2 hESCs (TRF3^−/−^-1+3Flag-TRF3 and TRF3^−/−^-2+3Flag-TRF3) and negative control ones (TRF3^−/−^-1+vector and TRF3^−/−^-2+vector).

### Immunocytochemical staining

Undifferentiated hESCs were stained using Alkaline Phosphatase (ALP) substrate kit III (Vector Laboratories, Burlingame, CA, USA) following the manufacturer’s instructions as previously described [4]. Immunocytochemical staining was carried out following the protocol described previously [12]. Briefly, harvest the attached cells at indicated stages, fix the cells with 4% paraformaldehyde (PFA), permeabilize the cells in 0.4% Triton X-100 (Sigma-Aldrich, Carlsbad, USA) for 20 min at room temperature to present the intracellular antigens (for membrane antigens, this step can be skipped), block the cells with 10% normal goat serum (Vector Laboratories, Burlingame, CA, USA), and then stain cells with antibodies against SSEA4 (stage-specific embryonic antigen 4) (cat no. MAB4304, Millipore, CA, USA, 1:200) and OCT4 (octamer-binding protein 3/4) (cat no. ab19857, Abcam, 1:200). The antibody labeling was visualized using DyLight 488/549-conjugated secondary antibodies (Jackson ImmunoResearch Laboratories, West Grove, PA, USA, 1:1000). Nuclei were stained with DAPI (2-(4-amidinophenyl)- 6-indolecarbamidine dihydrochloride) (Invitrogen, Carlsbad, CA, USA, 1 μg/ml). Slide observation and image capture were performed with a Zeiss Observer microscope.

### Flow cytometry analysis

The cells were handled as described previously [31]. Briefly, cells were harvested with Accutase (Stem Cell Technologies, Vancouver, Canada). For the detection of the membrane marker SSEA4, cells were fixed in 1% PFA and stained with FITC (fluorescein isothiocyanate)-conjugated SSEA4 antibody (cat no. 560126, BD Biosciences, San Jose, USA, 1:100) and isotype-matched controls. For the detection of the intracellular antigen OCT4, cells were fixed and permeabilized by Foxp3 Staining Buffer Set (Invitrogen, Carlsbad, CA, USA), blocked in Dulbecco’s phosphate-buffered saline (Gibco, Carlsbad, CA, USA) containing 5% FBS, then stained with primary un-conjugated OCT4 (cat no. ab19857, Abcam, 1:200) and followed by PE-conjugated secondary antibody (1:200; eBioscience, San Diego, USA). The cells incubated with secondary antibody only were used as negative controls. Cells were then analyzed and quantified with the flow cytometry (FACStar Plus Flow Cytometer, Becton-Dickinson, San Jose, CA, USA).

### Cell cycle analysis

Undifferentiated hESCs were prepared, fixed with 70% ethanol, and stained with 50 μg/ml PI (propidium iodide) as previously described [4]. Cell cycle distribution (G0–G1, S, and G2/M phases) were determined with flow cytometry (FACStar Plus Flow Cytometer, Becton-Dickinson).

### Quantitative reverse transcription polymerase chain reaction (qRT-PCR)

Total RNA was extracted with an RNAprep pure Micro Kit (TIANGEN, Beijing, PR China) and reverse transcribed with ReverTra Ace reverse transcriptase (Toyobo, Osaka, Japan) following the manufacturer’s instructions. Quantitative PCR was carried out and analyzed by the ViiA™ 7 Real-Time PCR System (Life Technologies, Carlsbad, CA, USA) with SYBR Green Q-PCR Master Mix (Roche, Mannheim, Germany). The qRT-PCR primers were listed in Additional file 2: Table S2. The qRT-PCR data were presented as fold changes normalized to internal control PBGD (porphobilinogen deaminase) [15].

### Western blot analysis

Sample preparation was carried out as reported [39, 62]. In brief, cells were collected, lysed in lysis buffer to get the whole cell lysate loaded for Western blots. Membranes with blots were incubated with the primary antibody against EOMES (cat no. ab23345, Abcam, 1:1000) and GAPDH (glyceraldehyde-3-phosphate dehydrogenase) (cat no. sc-47724, Santa Cruz Biotechnology, TX, USA, 1:5000). The membranes were then incubated with IRDye 680LT Donkey anti-Rabbit IgG (cat no. 926-68023, Li-COR Biosciences, Lincoln, NE, USA) or IRDye 800CW Donkey anti-Mouse IgG (cat no. 926-32212, Li-COR Biosciences, Lincoln, NE, USA) as secondary antibodies and visualized on an Odyssey Infrared Imager (Li-COR Biosciences, Lincoln, NE, USA).

### mRNA microarray analysis

Total RNA was extracted from three replicates of wild-type H1 hESCs (TRF3^+/+^) during the ME differentiation at day 0, day 1, day 2, and day 3 (hereafter as ME D0, ME D1, ME D2, ME D3) using Rneasy Mini Kit (Qiagen, Redwood City, CA, USA). The RNA samples were reverse transcribed, in vitro transcripted, and fragmented to fragmented and labeled amplified RNA (aRNA) using GeneChip 3′ IVT Express Kit (Santa Clara, CA, USA). The biotinylated aRNA was then hybridized to Affymetrix Gene Chip (Human Gene 1.0 ST arrays, Affymetrix). Twelve raw data files generated from the Affymetrix scanner passed data quality control and were further performed with RNA normalization through the Affymetrix expression console. All chips were normalized using the Robust Multi-array Average method implemented in Partek Genomics Suite 6.5 software. The signature mesendodermal, endoderm, and ectoderm genes were picked up, and the ratio was calculated by normalized signal value at each time point versus the values at day 0. The hierarchical average linkage clustering analysis was performed by using Cluster version 3.

### Chromatin immunoprecipitation (ChIP) and ChIP followed by massively parallel DNA sequencing (ChIP-seq) analysis

ChIP experiments were carried out with a Simple ChIP™ Enzymatic Chromatin IP Kit (cat no. 9002, Cell Signaling Technology, Danvers, MA, USA) following the manufacturer’s instructions. Protein-DNA complexes were immunoprecipitated with anti-FLAG® M2 affinity gel (cat no. A2220, Sigma-Aldrich, Carlsbad, USA) or Anti-FLAG M2 Magnetic Beads (catalog no. M8823, Sigma-Aldrich, Carlsbad, USA), and mouse IgG-agarose (cat no. A0919, Sigma-Aldrich, Carlsbad, USA). The ChIP-qPCR analysis was performed as previously described [10]. Briefly, the purified DNA was quantified by quantitative PCR with SYBR Green PCR reagents (Roche, Mannheim, Germany) to examine the enrichment of *T*, *EOMES*, *MIXL1*, and *GSC* genome with specific primers (listed in Additional file 3: Table S3) and normalized with the total input genome. For ChIP-seq, the purified DNA was sequenced by Shanghai Genefund Biotech Co Ltd (Shanghai, PR China) with Illumina Hi-Seq platform. Sequencing adapters, short reads (length < 35 bp), and low-quality reads were removed using Cutadapt (v1.18) and Trimmomatic (v0.38) [10] to obtain high-quality clean reads, which were also ensured by FastQC (http://www.bioinformatics.babraham.ac.uk/projects/fastqc/). Then, the clean reads were mapped to hg19 human reference genome with Bowtie2 software [37]. Peak calls were refined and reported by MACS (v2.1.2) (Model-based Analysis of ChIP-Seq) [67], and peak finding algorithm with 0.001 was set as the cutoff point of *p* value. Annotation of peak sites to gene features and the peak coverage picture plots were performed using the ChIPseeker R package [65]. The BigWig files with the subtracted input signal were generated using the function bamCompare in deeptools as reported previously [51], and then, the track screenshots of sequencing data were presented with the browser of Integrative Genomics Viewer (https://www.broadinstitute.org/igv/). Motif analyses were applied by MEME SUITE [5]. Quantitative comparison of ChIP-Seq data sets by 3Flag-TRF3 between ME D0 and ME D1 was inferred by MAnorm [54]. Functional enrichment analyses were implemented by clusterProfiler R package [64]. Gene regulatory networks were generated by Cytoscape software (http://www.cytoscape.org/) according to the gene ontology (GO) analysis results.

### Statistical analysis

The data were presented as mean ± SEM. Statistical significance of differences was estimated by one-way analysis of variance (ANOVA) followed with Bonferroni’s multiple analysis for qRT-PCR data from the ME differentiation samples of hESCs, and by two-way ANOVA followed with Tukey’s multiple comparison for the analysis of other qRT-PCR, Western blot, and ChIP-qPCR data. *p* < 0.05 was considered statistically significant.

## Results

### TRF3 is highly expressed during the ME differentiation of hESCs

**Fig. 1:**
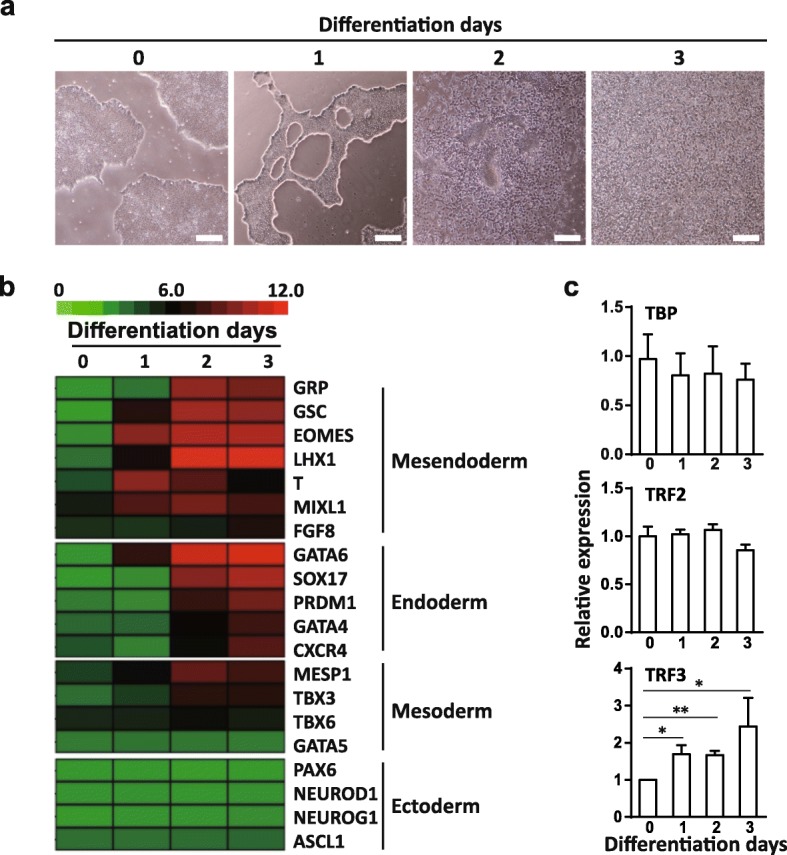
The TRF3 expression level is enhanced during ME differentiation of hESCs. **a** Microscope images for the morphology of ME differentiation. Scale bar = 200 μm. **b** The heat map of the expression pattern of early germ layer genes during the ME differentiation of hESCs based on the microarray analysis. The expression values in log2 scale were calculated and presented on the heat map with red representing highly abundant transcripts and green representing poorly abundant transcripts. *n* = 3 each. **c** qRT-PCR analysis of TBP, TRF2, and TRF3. Data are presented as mean ± SEM. *n* = 3 each. *p < 0.05, **p < 0.01 compared with the corresponding undifferentiated values

To explore the potential regulators for the ME differentiation of hESCs, the hESCs were induced into the ME differentiation as reported recently [4] following a modified monolayer differentiation protocol [15]. During the ME induction, the colony shape of hESCs gradually disappeared and the cells migrated out to form the uniform layer (Fig. 1a). Microarray analysis revealed the upregulation of ME signature genes EOMES, T, MIXL1, and GSC, while the expression of early neuroectodermal marker genes paired box 6 (PAX6), neuronal differentiation 1 (NEUROD1), neurogenin 1 (NEUROG1), and achaete-scute family BHLH transcription factor 1 (ASCL1) remained unchanged (Fig. 1b). These data suggest that hESCs are successfully induced into the ME cells as previously reported by Chng et al. [15] and us [4]. By examining the expression of TBP family members, we found that TRF3 was significantly upregulated during the ME differentiation of hESCs, while no significant changes were observed in the expression levels of TBP and TRF2 (Fig. 1c), indicating that TRF3 might be involved in the ME differentiation of hESCs.

### Depletion of TRF3 does not affect hESC self-renewal

**Fig. 2:**
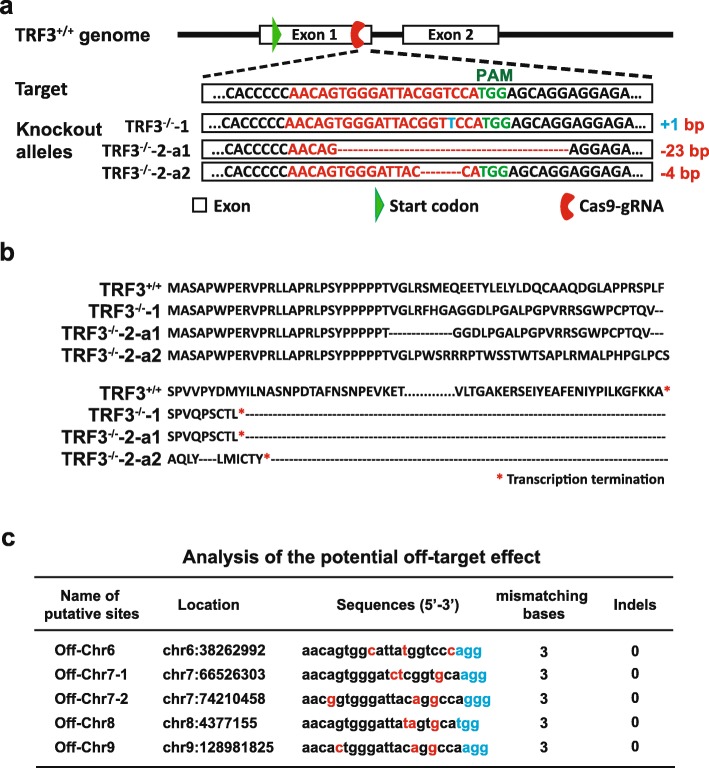
Generation of TRF3 knockout (TRF3^−/−^) hESCs. **a** Targeting strategy for the generation of TRF3^−/−^ hESCs by homologous recombination using CRISPR/Cas9 technology. **b** Translation analysis of TRF3 truncated proteins in TRF3^+/+^, TRF3^−/−^-1, and TRF3^−/−^-2 hESCs. a1/2, the TRF3 genome sequence of each allele in TRF3^−/−^-2 hESCs. **c** Potential off-target sites predicted by CHOPCHOP. The predicted off-target sites are in the “Name of putative sites” column. The mismatched bps are shown in red. The protospacer adjacent motif is shown in cyan. Indels: the confirmed mutant bps at each potential off-target site

To determine whether TRF3 regulates hESC fate decision, we established two TRF3 knockout hESC lines (TRF3^−/−^-1 and TRF3^−/−^-2) from wild-type H1 hESCs (TRF3^+/+^) by CRISPR/Cas9 (Fig. 2a). The insertions/deletions (indels) were confirmed by Sanger sequencing with the primers listed in Additional file 1: Table S1. The primers located at 350 base pairs (bps) upstream and 280 bps downstream of the cleavage site. The Sanger sequencing revealed that the two TRF3^−/−^ cell lines (TRF3^−/−^-1 and TRF3^−/−^-2) had different indels. The TRF3^−/−^-1 cell line had 1 “T” insertion while the TRF3^−/−^-2 cell line had 23 bp and 4 bp deletions at each allele (Fig. 2a). Considering the fact that most of the deletions, generated by error-prone non-homologous end-joining after the cleavage by Cas9, are small ones (varies from 1 bp to several dozen bps) and the homozygous mutant clones can occur in hPSCs as previously reported [13, 23, 27, 60], the mutation in TRF3^−/−^-1 hESCs appears to be the bi-allelic + 1 insertion [60]. Further, we examined the five predicted off-target sites in the TRF3^−/−^ cells using Sanger sequencing. None of the five sites in either of the TRF3^−/−^-1 or of the TRF3^−/−^-2 clones was mutated (Additional file 4: Figure S1).

**Fig. 3:**
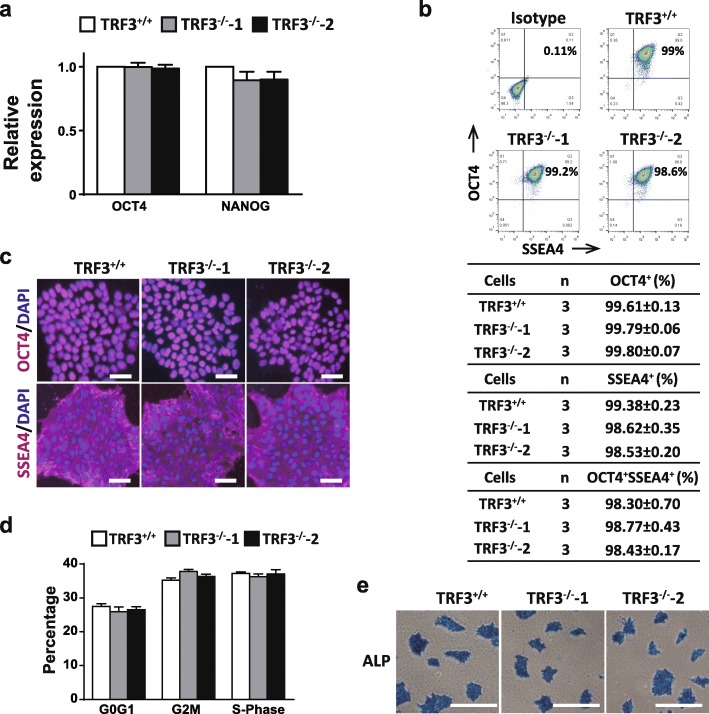
The expression levels of pluripotent markers are comparable among the undifferentiated TRF3^+/+^, TRF3^−/−^-1, and TRF3^−/−^-2 hESCs. **a** qRT-PCR analysis of pluripotency markers OCT4 and NANOG. *n* = 3. **b** Flow cytometry analysis of OCT4 and SSEA4. **c** Immunocytochemical staining analysis of OCT4 and SSEA4. Similar results were obtained from three independent experiments. Scale bar = 50 μm. **d** Flow cytometry analysis of cell cycle in undifferentiated hESCs. *n* = 3. **e** Representative analysis of clones positive for alkaline phosphatase staining. Similar results were obtained from three independent experiments. Scale bar = 500 μm. Data are presented as mean ± SEM

The indels in TRF3 locus resulted in the early termination of translations (Fig. 2b). Next, we tested the self-renewal and pluripotency markers in TRF3^+/+^ and TRF3^−/−^ hESCs. The expression levels of pluripotency marker genes OCT4 and NANOG (Fig. 3a) were comparable between the undifferentiated TRF3^+/+^ and TRF3^−/−^ hESCs. The similar phenomena in the protein levels of OCT4 and SSEA4 were confirmed by flow cytometry (Fig. 3b) and immunocytochemical staining (Fig. 3c). In addition, the cell cycle distribution (Fig. 3d) and the ALP activity (Fig. 3e) were similar between the undifferentiated TRF3^+/+^ and TRF3^−/−^ hESCs. These results suggest that TRF3 may not play important roles in undifferentiated hESCs.

### Depletion of TRF3 inhibits the ME lineage but not neuroectodermal commitment of hESCs

**Fig. 4:**
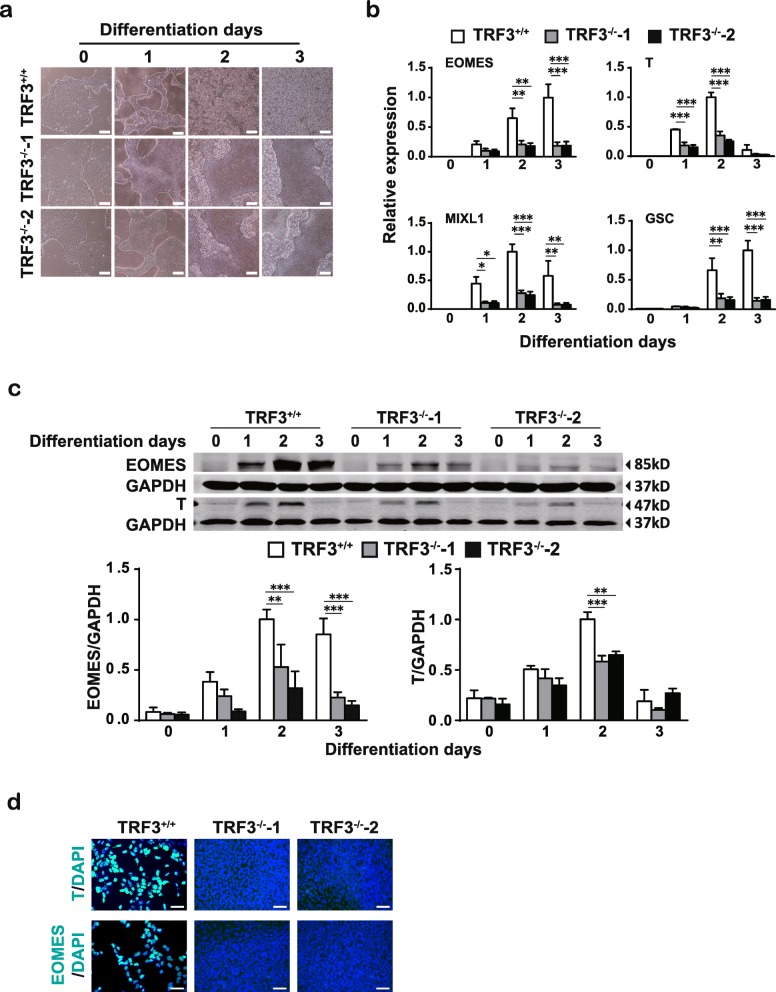
Depletion of TRF3 inhibits the ME differentiation of hESCs. **a** Cell morphology analysis of TRF3^+/+^, TRF3^−/−^-1, and TRF3^−/−^-2 hESCs during the ME differentiation. Scale bar = 200 μm. **b** qRT-PCR analysis of ME markers during the ME differentiation of TRF3^+/+^, TRF3^−/−^-1, and TRF3^−/−^-2 hESCs. **c** Western blot analysis of EOMES, T, and GAPDH. GAPDH was used as a loading control. **d** Immunocytochemical staining analysis of T and EOMES at ME D2 in TRF3^+/+^, TRF3^−/−^-1, and TRF3^−/−^-2 hESCs. Scale bar = 25 μm. Data are presented as mean ± SEM. *n* = 3 each. **p < 0.01, ***p < 0.001 as indicated on the figures

To determine the role of TRF3 in ME differentiation, we induced hESCs into ME. During the ME differentiation, the cell morphology differed between the TRF3^+/+^ and TRF3^−/−^ hESCs (Fig. 4a). Most of the cells lost their clone-like shape and spread in the culture dish at differentiation day 3 in TRF3^+/+^ cells, while the large scales of TRF3^−/−^ cells remained a clone-like morphology (Fig. 4a), suggesting the obstruction of ME differentiation after TRF3 deficiency. Indeed, qRT-PCR analysis showed that the expression of the key ME transcription factor genes, *EOMES*, *T*, *MIXL1*, and *GSC* in the TRF3^+/+^ cells, was largely suppressed in the TRF3^−/−^ cells during ME differentiation (Fig. 4b). The downregulation of EOMES and T in the TRF3^−/−^ cells was confirmed by Western blot (Fig. 4c). The immunocytochemical staining further confirmed that the high proportions of cells positive for T and EOMES were detected at ME D2 in TRF3^+/+^ cells, but they were hardly detected in TRF3^−/−^ cells (Fig. 4d). Consistent with the observations in Fig. 4a, the TRF3^+/+^ cells at ME D2 lost the colony morphology and spread out, while the TRF3^−/−^ cells remained crowded (Fig. 4d). These data indicate that TRF3 deficiency significantly impedes the ME differentiation from hESCs.

To determine the identity of the TRF3^−/−^ cells during the ME differentiation, we analyzed the pluripotent and neuroectodermal markers. At the ME D3, the expression of OCT4 and NANOG became much lower than these at day 0 in both TRF3^+/+^ and TRF3^−/−^ cells (Additional file 5: Figure S2a), though the OCT4 level in the TRF3^−/−^ cells was significantly higher than that in the TRF3^+/+^ cells. In addition, the expression of neuroectodermal genes GBX2 and NEUROD1 did not show significant alterations during the ME differentiation, while the expression of SOX1 was mildly enhanced in the TRF3^−/−^-1 cells but remained unchanged in the TRF3^−/−^-2 cells (Additional file 5: Figure S2b). Taken together, the TRF3^−/−^ cells loss their pluripotency during the ME differentiation, though some of them remain a clone-like morphology; the TRF3^−/−^ cells at ME D3 are neither undifferentiated, ME, or neuroectodermal cells.

To further determine whether the TRF3 deficiency influences early neuroectodermal lineage commitment, we used a monolayer neuroectodermal differentiation protocol of hESCs modified from previously reported [4, 15]. During the neuroectodermal induction, the TRF3 expression level was slightly downregulated at the early phase and then was upregulated at the late phase of neuroectodermal differentiation (Additional file 5: Figure S2c). qRT-PCR analysis did not show significant differences in the mRNA levels of pluripotency genes OCT4 and NANOG (Additional file 5: Figure S2d) and early neuroectodermal genes SOX1, SOX2, SIP1, SIX1, GBX2, and NEUROD1 (Additional file 5: Figure S2e) between the TRF3^+/+^ and TRF3^−/−^ cells. Taken together, these data suggest that TRF3 is not required for early neuroectodermal differentiation.

### Reintroduction of TRF3 into TRF3^−/−^ hESCs rescues ME differentiation of hESCs

**Fig. 5:**
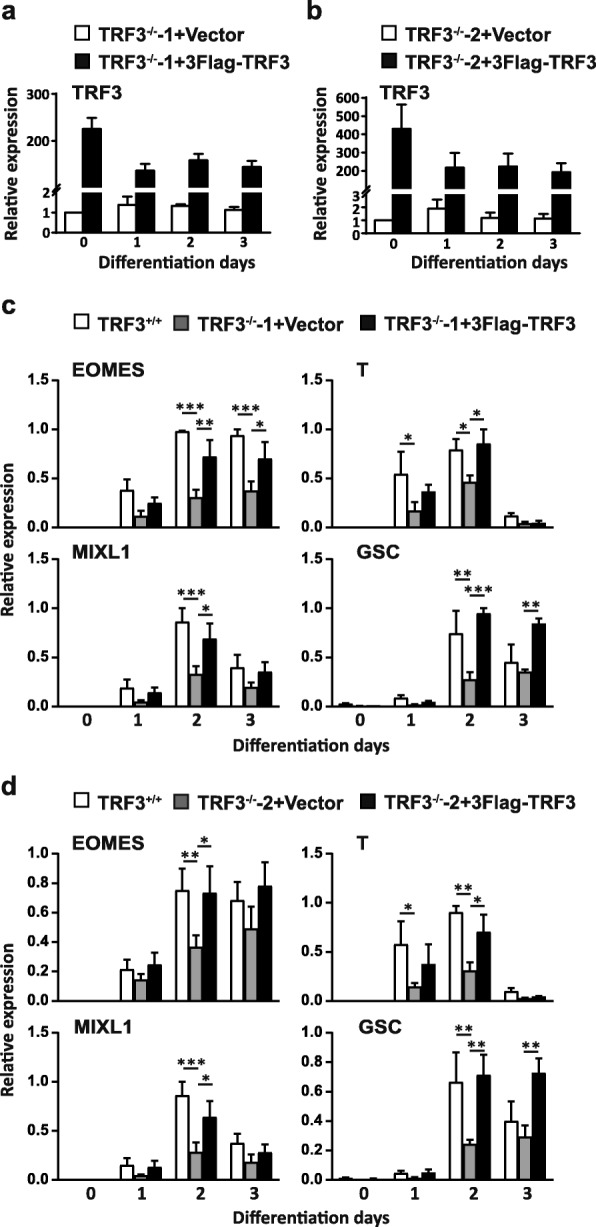
Reintroduction of TRF3 into TRF3^−/−^-1 and TRF3^−/−^-2 hESCs rescues the ME differentiation. **a** qRT-PCR analysis of TRF3 in the TRF3^−/−^-1+3Flag-TRF3 and TRF3^−/−^-1+vector control cells. *n* = 3 each. **b** qRT-PCR analysis of TRF3 in the TRF3^−/−^-2+3Flag-TRF3 and TRF3^−/−^-2+vector control cells. *n* = 3 each. **c** qRT-PCR analysis of the ME markers in the TRF3^+/+^, TRF3^−/−^-1+vector control, and TRF3^−/−^-1+3Flag-TRF3 hESCs during the ME differentiation. *n* = 3 each*.***d** qRT-PCR analysis of the ME markers in the TRF3^+/+^, TRF3^−/−^-2+vector control, and TRF3^−/−^-2+3Flag-TRF3 hESCs during ME differentiation. *n* = 4 each*.* Data are presented as mean ± SEM. *p < 0.05, **p < 0.01, ***p < 0.001 compared with the corresponding vector overexpressing values

To verify the contribution of TRF3 to the ME fate decision of hESCs, we transfected two TRF3^−/−^ hESC lines with the lentiviruses containing 3Flag-TRF3 and the lentiviruses with puromycin resistance gene alone as a control. Four cell lines were generated after puromycin selection, named TRF3^−/−^-1+vector, TRF3^−/−^-1+3Flag-TRF3, TRF3^−/−^-2+vector, and TRF3^−/−^-2+3Flag-TRF3. qRT-PCR analysis confirmed the successful overexpression of TRF3 in the TRF3^−/−^-1 (Fig. 5a) and TRF3^−/−^-2 (Fig. 5b) hESCs. The transfected cells represented colony morphology and positive for ALP activity (Additional file 6: Figure S3a). The expression of pluripotency markers (*OCT4* and *NANOG*, Additional file 6: Figure S3b) and mesendodermal signature genes (*EOMES*, *T*, *MIXL1*, *GSC*, Additional file 6: Figure S3c) were comparable between TRF3^+/+^, TRF3^−/−^+vector, and TRF3^−/−^+3Flag-TRF3 cells at undifferentiated status. During the ME differentiation, the restoration of TRF3 in the TRF3^−/−^ hESCs rescued the differentiation of ME in both TRF3^−/−^-1+3Flag-TRF3 and TRF3^−/−^-2+3Flag-TRF3 cells, as indicated by the recovery of mRNA expression of signature genes (*EOMES*, *T*, *MIXL1*, and *GSC*) (Fig. 5c, d); the expression of ME genes in the TRF3^−/−^-1+3Flag-TRF3 and TRF3^−/−^-2+3Flag-TRF3 cells were comparable with those in the TRF3^+/+^ cells. These results indicate that TRF3 can promote the expression of ME lineage genes with ME inducible signals but not in the undifferentiated status.

### TRF3 regulates ME lineage commitment by activating the transcription of ME genes

**Fig. 6:**
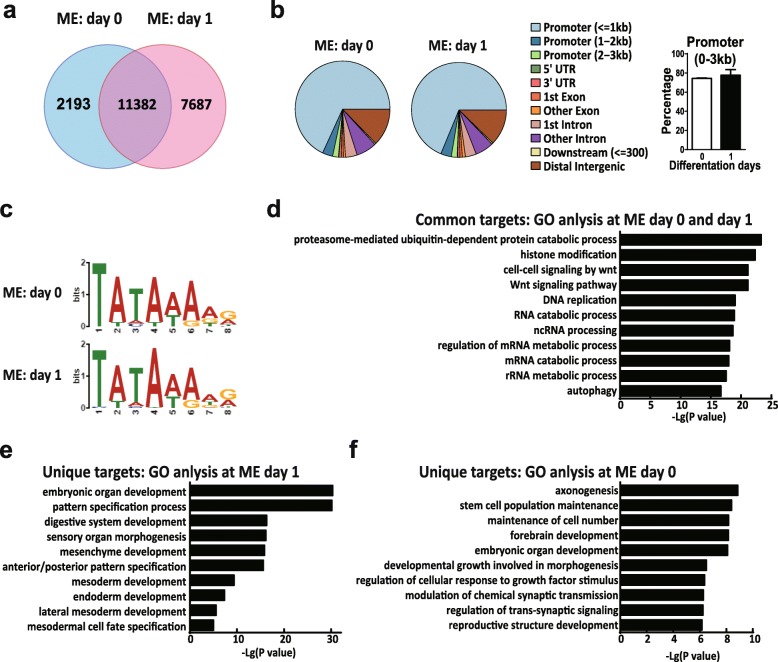
ChIP target analysis of TRF3 at the undifferentiated status (ME D0) and the ME D1. **a** ChIP-seq data overlaps between the ME differentiation day 0 and day 1. **b** Peak distribution of ChIP-seq data (left panel) on the genome and quantitative analysis of TRF3 bindings at promoter regions (right panel, *n* = 3). **c** Motif analyses of ChIP-seq data. Similar results were obtained from three independent experiments. **d**–**f** Gene ontology analyses of ChIP-seq data

To understand the mechanism of TRF3 in the regulation of ME specification from hESCs, we performed ChIP-seq to determine the genome-wide binding of TRF3 at the undifferentiated status (day 0) and the early ME stage (differentiation day 1). As shown in Fig. 6a, the quantitative comparison of the ChIP-Seq data sets between the ME D0 and D1 revealed that the target genes of TRF3 can be divided into 3 groups: uniquely bound by TRF3 at day 0 (U0), uniquely bound by TRF3 at the ME D1 (U1), and the genes bound by TRF3 in both stages, i.e., common targets. There were 11,382 common binding targets of TRF3, 2193 targets unique at ME D0, and 7687 targets unique at ME D1 (Fig. 6a). The amount of U1 genes was almost 3.5 times larger than U0 genes, suggesting a significant change in the binding profile. The peak distribution analysis showed that TRF3 mainly bound to the promoter areas of target genes (more than 70%), no matter at differentiation day 0 or at differentiation day 1 (Fig. 6b). The analysis of mean peak counting frequency flanking transcription start site (TSS) regions represented the similar pattern (Additional file 7: Figure S4a) with the TBP ChIP-seq data as reported previously [40]. The motif analyses further showed that TRF3 mainly recognized the “TATA” sequence (Fig. 6c), which was consistent with previous reports [33]. Gene ontology (GO) enrichment analyses exhibited that the common targets mainly enriched for histone modification, DNA replication, metabolic processes, and so on (Fig. 6d). These data indicate that TRF3 may have a fundamental function in the hESC maintenance and during the ME differentiation process. The U1 genes were significantly enriched in functions regulating mesodermal and endodermal development and formation (Fig. 6e), which demonstrates an extensive regulation targets of TRF3 in ME specification. Although the unique ME D0 targets were mainly for the stem cell maintenance and cellular response to growth factor stimulus and synaptic signaling pathways (Fig. 6f), the morphology of TRF3^−/−^ hESCs was similar as that of the TRF3^+/+^ control hESCs (Fig. 3) (see the “Discussion” section). Taken together, these ChIP-seq data demonstrates a large regulatory spectrum of TRF3 in the ME specification.

**Fig. 7:**
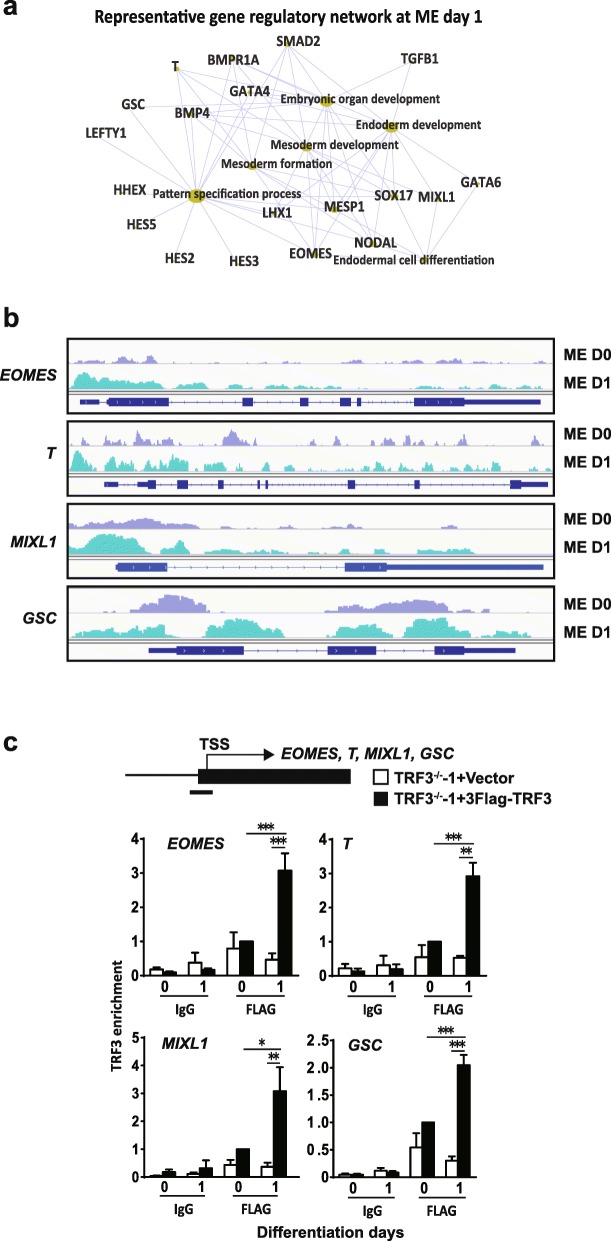
ChIP analyses of TRF3 at the promoter regions of ME markers at undifferentiated status (ME D0) and ME D1 induction of hESCs. **a** Gene concept network displaying the representative gene names associated with differentiation or development at ME D1. **b** Genome browser screenshots of ChIP-seq for *T*, *EOMES*, *MIXL1*, and *GSC* at the ME D0 and day 1. **c** ChIP-qPCR analyses of TRF3 at the transcription start sites (TSS) of ME markers (*EOMES*, *T*, *MIXL1*, and *GSC*) in TRF3^−/−^-1+vector and TRF3^−/−^-1+TRF3 hESCs. Data are presented as mean ± SEM. *n* = 3 each*.* *p < 0.05, **p < 0.01, ***p < 0.001 compared with the corresponding vector overexpressing values at differentiation day 1

Next, we performed gene concept network analyses to explore the detailed regulation network of TRF3 in ME specification. As shown in Fig. 7a, some key ME genes emerged as the targets of TRF3, such as *EOMES*, *T*, *MIXL1*, and *GSC*. Genome browser screenshots of ChIP-seq showed the enhancement of TRF3 on the promoter region of *EOMES*, *T*, and *MIXL1* at ME D1 compared with those at the undifferentiated status (ME D0) (Fig. 7b). Interestingly, the enrichment of TRF3 at GSC locus showed different binding patterns between ME D0 and D1. At ME D0, TRF3 mainly bound to part of the first exon, the following exons, and the second intron, while at ME D1, TRF3 bound to the promoter, exons, and part of the first and second introns, indicating that there might be another regulatory way of TRF3 in the regulation of TRF3 in ME gene expression. In contrast, on the promoters of neuroectodermal genes, such as *SOX1*, *NEUROD*, *NEUROG*, *PAX6*, and *SIP1* (used as negative control genes here), TRF3 showed low and unchanged enrichment of TRF3 at ME D1 versus that at ME D0 (Additional file 7: Figure S4b). The enrichment of TRF3 on pluripotent markers *OCT4* and *NANOG* was consistent with the previous reports that both of the two genes are necessary for the early mesendodermal lineage differentiation [22, 25, 41, 57]. Further, ChIP-qPCR analysis confirmed that the bindings between the TRF3 and the TSS of ME transcription factor genes *EOMES*, *T*, *MIXL1*, and *GSC* in the TRF3^−/−^-1+3Flag-TRF3 hESCs were enhanced after 1 day of ME induction, while this was not detected in the TRF3^−/−^-1+vector control cells (Fig. 7c).

These data, together with the observations of the downregulation of the key ME gene levels in the TRF3^−/−^ cells (Fig. 4), and the restoration by the reintroduction of TRF3 into the TRF3^−/−^ cells (Fig. 5), support that TRF3 contributes to the ME lineage specification.

## Discussion

In the present study, using an in vitro hESC ME differentiation model, we discover that TRF3 but not TBP or TRF2 is significantly increased during the ME commitment of hESCs. Based on the CRISPR technology and ChIP-seq analysis, we have identified that (i) the depletion of TRF3 does not affect the hESC self-renewal; (ii) during the mesendodermal differentiation, TRF3 deficiency suppresses the mesendodermal differentiation but does not affect the neuroectodermal differentiation of hESCs; (iii) the global binding profile of TRF3 shifts from the genes mediating hESC maintenance and growth to the genes determining cell fate during the ME specification; and (iv) TRF3 directly binds to the core promoters of key ME signature genes, *EOMES*, *T*, *MIXL1*, and *GSC*, to initiate their transcriptions. These findings identify TRF3 as a critical component for directing cell lineage specification and reveal a novel role of TRF3 in the regulation of germ layer decision of hESCs.

One finding here is the determination of the expression pattern of TRF3 during human ME differentiation. The expression pattern of TRF3 in human ME differentiation is consistent with previous reports that the expression of TRF3 is upregulated during early embryo development of zebrafish and differentiation of mESCs in an embryoid body model [6, 29]. These observations suggest that TRF3 may have an important role in the early development. Notably, the elevated level of TRF3 is mild during the ME differentiation. However, the TRF3 knockout (Fig. 4) and rescue (Fig. 5), ChIP-seq (Figs. 6 and 7), and ChIP-qPCR (Fig. 7) data confirmed that TRF3 does promote the expression of ME lineage genes. The mild change of TRF3 expression level during the ME differentiation could be interpreted by the different working model of different transcription factors in the promoting of ME specification. The key mesendodermal transcription factors, such as T and EOMES, play a crucial role in “guiding” pluripotent cells to the ME lineage commitment [59], while TRF3 appears to play a role in helping to promote the expression of ME lineage genes. Supportively, the overexpression of TRF3 in TRF3^−/−^ cells does not affect the expression of self-renewal and pluripotent markers (Additional file 6: Figure S3), indicating that TRF3 cannot initiate the ME gene expression without inducing signals.

It is noteworthy that TRF3 depletion in the zebrafish and mouse has distinct phenotypes, despite that TRF3 promotes the ME differentiation of hESCs. In zebrafish, the TRF3-knockdown embryos using morpholino oligonucleotide exhibit delayed development by 14 h post-fertilization [28], while the TRF3-deficient mice are viable without apparent phenotypes [26]. In our study, TRF3^−/−^ cells show strong deficiency in the ME differentiation of hESCs. Thus, TRF3 exhibits species-dependent function. One possible explanation for this complexity is that the N-terminal of TRF3 is highly divergent among different species [6, 49]. Unlike the highly conserved C-terminal core domain of TRF3, which is more than 90% conserved between human and mouse or zebrafish, the N-terminal is only 47.8% conserved between human and mouse and 21.3% conserved between human and zebrafish (Additional file 4: Figure S2). Therefore, the non-conserved N-terminal domain of TRF3 might confer the species-dependent function of TRF3 in early development. To further test this hypothesis, mutated forms of TRF3, such as TRF3 carrying the dead DNA binding domain, TRF3 truncations without its DNA binding domain, or TRF3 truncations without its N-terminal domain, could help to understand the mechanism of TRF3 in the regulation of ME differentiation.

Another important finding here is the revelation of the global binding profile of TRF3. TRF3 has been shown to bind to the promoter region of specific genes such as *mespa* and Myogenin [19, 28]; however, the global binding profile of TRF3 is unknown. Comparing the ChIP-seq data in the ME D1 with ME D0, we found a significant shift of TRF3 binding pattern. As shown in Fig. 6, the genes uniquely bound by TRF3 at the ME D1 are highly enriched in mesendoderm specification, which indicates that TRF3 binds to the promoters of numerous ME genes during the ME differentiation. Interestingly, GO analysis of genes bound by TRF3 at the ME D0 reveals that TRF3 mainly binds to genes involved in the proteasome-dedicated ubiquitin-dependent protein catabolic process, histone modification, DNA replication and RNA processing, and stem cell population maintenance at hESC state (ME D0), suggesting that TRF3 might also regulate the maintenance of hESCs. However, this is inconsistent with the data that the TRF3 deficiency does not significantly affect cell cycle distributions and the expression levels of pluripotency markers (Fig. 3). These conflicted results might be explained by the functional redundancy of TBP or TRF2. It has been shown that TRF3 could partially substitute for TBP [33], and TBP might be functional redundancy vice versa*.* However, whether TBP or TRF2, to some extent, substitutes TRF3 at the hESC stage needs to be tested in the future.

The specific bindings of TRF3 to the ME genes were supported by the following data: (i) most of the reads locate in the proximal promoter region (promoter ≤ 1 kb) (Fig. 6b), which is consistent with the fact that TRF3 is a transcription factor [2, 45]; (ii) the motif analysis of ChIP-seq data demonstrates the specific binding of TRF3 to the TATA box site; (iii) the mean peak counting frequency flanking TSS (Additional file 7: Figure S4a) is similar to the TBP ChIP data reported previously [36, 40]; (iv) the genome browser screenshots of neuroectodermal genes, which were used as negative control genes, are in a low and unchanged enrichment between day 0 and day 1 (Additional file 7: Figure S4b), rather than the increased TRF3 enrichment in the promoter region of mesemdodermal genes (Fig. 7b); (v) the ChIP-qPCR data confirms more TRF3 bindings to the promoter regions of key mesendodermal genes at day 1 versus these at day 0 (Fig. 7c); and (vi) the knockout and rescue of TRF3 confirm the promotive effect of TRF3 in the ME differentiation. However, considering the ChIP-seq is done in cells with TRF3 overexpression, the bindings of TRF3 at physiological level need to be elucidated further.

Our ChIP-seq data also suggests that TRF3 can bind to the other sequences in genome, such as the exons, introns, and distal intergenic regions, revealed by ChIP-seq (Fig. 6b). However, the function of the atypical binding pattern, with a low percentage among the total distribution, is unknown. Different with the binding of TRF3 at the promoter region of the *EOMES*, *T*, and *MIXL1* locus, the genome browser screenshots reveal that TRF3 can bind to the first exon and one intron at ME D0 and shift to the promoter and exon region of *GSC* locus at ME D1 (Fig. 7b). The binding of TRF3 with GSC genome sequences at ME D0 without initiation of the transcription (Additional file 6 Figure S3c) suggests that TRF3 might involve in other functions rather than direct transcription initiation. The transcription factors EOMES and T are recently demonstrated to modulate the chromatin accessibility by binding to the introns and distal intergenic regions [59], which suggests that transcription factors might function beyond the special transcription initiation. However, this possibility needs to be investigated in the future.

In addition, our data prove that TRF3 can specifically promote the expression of ME genes, but the molecular mechanism for the selectivity of TRF3 is not fully uncovered. As discussed above, TRF3 cannot initiate the expression of ME genes when lacking of inducing signals, indicating that it may work as an executor to regulate downstream gene expression following the differentiation signals, rather than a modulator to regulate the executor protein to initiate ME gene expression. Supportively, as revealed by the ChIP-seq data, the TRF3 DNA binding profile is significantly shifted in the ME D1 compared with the ME D0. Transcription initiation in eukaryotic cells is an exceedingly intricate process that requires the precise orchestration of a complex set of interactions between a myriad of trans-acting factors (proteins) and cis-acting elements (DNA sequences) [43]. The expression of lineage-specific genes relies on the interactions among tissue-specific enhancers, transcription factors, and epigenetic regulators, all of which cooperate with the general transcription factors to initiate the gene expression [24]. Given the specific binding motif revealed by our ChIP-seq data, the binding specificity of TRF3 might be guided by other proteins. In myofibroblast, MyoD can interact with TAF3/TRF3 to activate the Myogenin transcription to promote myogenesis [19–21], which is similar with our hypothesis. The interaction of TRF3; ME differentiation signals, such as SMADs and β-catenin; and other ME-specific transcription factors requires further studies.

## Limitation

As the lack of qualified TRF3 antibodies, we could not validate the absence of TRF3 protein in the TRF3^−/−^ cells. Although the Sanger sequencing and ME differentiation analysis of TRF3 knockout and rescue experiments confirm the deficiency of TRF3 and its crucial role in promoting the expression of ME lineage genes, it would be better to confirm the protein level of TRF3 in the TRF3^−/−^ hESCs. Considering CRISPR/Cas9 has been used in the precise knockin in mouse embryos and hPSCs [69], building a cell line to tag the endogenous TRF3 by CRISPR/Cas9 can be used as an alternative approach to help figuring out the expression pattern at protein level and needs to be done in the future.

## Conclusions

Our study reveals a novel mechanism of human ME lineage commitment, i.e., the general transcription factor TRF3 specifically recognizes the TATA box within the promoter regions of ME genes, especially the four key transcription factors (*T*, *EOMES*, *MIXL1*, and *GSC*) to initiate ME differentiation of hESCs. These findings uncover a unique role of TRF3 in the early lineage fate decision of hESCs and a novel mechanism of the global gene regulatory network shift directed by a general transcription factor. These findings, together with other reports [3, 53, 59], indicate that the early lineage fate determination is under multi-dimensional and tight control to guarantee the precise lineage specification.

## Supplementary information

**Additional file 1: Table S1.** Primer sequences for mutation detection.

**Additional file 2: Table S2.** Primer sequences for qRT-PCRs.

**Additional file 3: Table S3.** Primer sequences for ChIP-qPCRs.

**Additional file 4: Figure S1.** Sanger sequencing of the predicted off-target sites. The name of predicted off-target sites locates above each panel. The protospacer adjacent motif is indicated by short cyan lines. The red arrow heads and dashed rectangles indicate the single nucleotide polymorphism.

**Additional file 5: Figure S2.** qRT-PCR analysis of pluripotency and neuroectodermal markers during the ME and neuroectodermal differentiation. (a): the expression of pluripotency markers (OCT4, NANOG) during the ME differentiation. *n* = 3 each. *p < 0.05, **p < 0.01, ***p < 0.001 as indicated. (b): the expression of neuroectodermal markers (SOX1, GBX2 and NEUROD1) during the ME differentiation. *n* = 3 each. *p < 0.05, compared with the corresponding values in undifferentiated cells. n.s., no significant difference compared with the corresponding values in undifferentiated cells. (**c**): qRT-PCR analysis of TRF3 during the neuroectodermal differentiation process of TRF3^+/+^ hESCs. (**d**): qRT-PCR analysis of pluripotency markers (OCT4, NANOG). *n* = 3 each. (**e**): qRT-PCR analysis of neuroectodermal markers (SOX2, SOX1, SIP1, SIX1, GBX2 and NEUROD1). *n* = 3 each. Data are presented as mean ± S.E.M.

**Additional file 6: Figure S3.** Reintroduction of TRF3 into TRF3^-/-^-1 and TRF3^-/-^-2 hESCs do not affect the self-renewal and the expression of ME genes in undifferentiated status. Reintroduction of TRF3 into TRF3^-/-^- hESCs does not affect the self-renewal and the expression of ME genes in the undifferentiated status. (a): Cell morphology and ALP activity of TRF3^-/-^-1, TRF3^-/-^-1+Vector, TRF3^-/-^-1+3Flag-TRF3, TRF3^-/-^-2, TRF3^-/-^-2+Vector, TRF3^-/-^-2+3Flag-TRF3 cells. Scale bar = 100 μm. (b): qRT-PCR analysis of pluripotency markers (OCT4 and NANOG) in TRF3^+/+^, TRF3^-/-^-1+Vector, TRF3^-/-^-1+3Flag-TRF3, TRF3^-/-^-2+Vector, TRF3^-/-^-2+3Flag-TRF3 hESCs. *n* = 3 each. (c): qRT-PCR analysis of ME genes (EOMES, T, MIXL1 and GSC) in TRF3^+/+^, TRF3^-/-^-1+Vector, TRF3^-/-^-1+3Flag-TRF3, TRF3^-/-^-2+Vector, TRF3^-/-^-2+3Flag-TRF3 hESCs. *n* = 3 each.

**Additional file 7: Figure S4.** ChIP analysis of TRF3 at undifferentiated status (ME D0) and ME D1. (a): The mean peak counting frequency flanking TSS in the undifferentiated status (ME D0) and ME D1. (b): Genome browser screenshots of ChIP-seq for pluripotency markers (*OCT4* and *NANOG*) and neuroectodermal genes (*SOX1, NEUROD, NEUROG, PAX6, SIP1*) in the undifferentiated status (ME D0) and ME D1.

**Additional file 8: Figure S5.** Amino acid sequence analysis of TRF3 proteins among human, mouse and zebrafish. (**a**): Amino acid sequence analysis of TRF3 protein. (**b**): Sequence conservation analysis of TRF3.

## Data Availability

Data and materials used and/or analyzed during the current research are available from the corresponding author on reasonable request.

## Abbreviations

TBP: TATA box-binding protein
PIC: Preinitiation complex
hESCs: Human embryonic stem cells
CRISPR: Clustered regularly interspaced short palindromic repeats
TRF3: TBP-related factor 3
ChIP: Chromatin immunoprecipitation
TRF3^−/−^: TRF3 knockout
ME: Mesendoderm
EOMES: EOMESODERMIN
T: BRACHYURY
MIXL1: Mix paired-like homeobox
GSC: GOOSECOID homeobox
GTFs: General transcription factors
WNT: Wingless-type MMTV integration site family
hPSCs: Human pluripotent stem cells
TRF1: TBP-related factor 1
TBPL2: TBP like 2
CDM: Chemically defined medium
BMP4: Bone morphogenetic protein 4
bFGF: Basic fibroblast growth factor
Cas9: CRISPR associated 9
ROCK: Rho-associated coiled-coil kinase
ALP: Alkaline phosphatase
SSEA4: Stage-specific embryonic antigen 4
OCT4: Octamer-binding protein 3/4
FITC: Fluorescein isothiocyanate
PI: Propidium iodide
qRT-PCR: Quantitative reverse transcription polymerase chain reaction
PBGD: Porphobilinogen deaminase
GAPDH: Glyceraldehyde-3-phosphate dehydrogenase
WT: Wild-type
ChIP-seq: ChIP followed by massively parallel DNA sequencing
ANOVA: Analysis of variance
PAX6: Paired box 6
NEUROD1: Neuronal differentiation 1
NEUROG1: Neurogenin 1
ASCL1: Achaete-scute family BHLH transcription factor 1
indels: Insertions/deletions
bp: Basepair
SOX1: Sex determining region Y-Box 1
SIP1: Smad-interacting protein 1
SIX1: SIX homeobox 1
GBX2: Gastrulation brain homeobox 2
GO: Gene ontology
TSS: Transcription start sites
