# Supplementary material for: TATA box-binding protein-related factor 3 drives the mesendoderm specification of human embryonic stem cells by globally interacting with the TATA box of key mesendodermal genes
Source: Stem Cell Res Ther. 2020 May 24;11:196. doi: 10.1186/s13287-020-01711-w (PMC7245780; doi:10.1186/s13287-020-01711-w)
Supplement: Supplementary file 1 — Additional file 1: Table S1. Primer sequences for mutation detection. [file 13287_2020_1711_MOESM1_ESM.docx]

**Additional file 1 Table S1. Primer sequences for mutation detection**

| Names | Primer sequences (5’-3’) | AT (ºC) | PS (bps) |
| --- | --- | --- | --- |
| TRF3-seq | F- GGAGCAAAGACCACATTGCG  R- AACGGTTCTGGATTCGTGCT | 60 | 635 |
| Off-Chr6-seq | F- TACCAGGGGTTCAGTTTACAGC  R- ATTAGCCCAGCATGGATTGGG | 60 | 968 |
| Off-Chr7-1-seq | F- AAAAGCCCCCAATGCAGAGT  R- GGCTTCCATGGGAACCTTCA | 60 | 996 |
| Off-Chr7-2-seq | F- GTGGTCCCATCATTGCCAGA  R- CTCTGCTCTGGGGTCTGTTG | 60 | 524 |
| Off-Chr8-seq | F- GTTCACTCCTCCACTCACCG  R- GCTTCTTCGCAAAGTCCAGC | 60 | 640 |
| Off-Chr9-seq | F- CCTGGCATCATCACCACTGA  R- TGGTTTGACCCCCTGAGAGA | 60 | 906 |

F, forward; R, reverse; AT, annealing temperature; PS, product size.
