# Supplementary material for: TATA box-binding protein-related factor 3 drives the mesendoderm specification of human embryonic stem cells by globally interacting with the TATA box of key mesendodermal genes
Source: Stem Cell Res Ther. 2020 May 24;11:196. doi: 10.1186/s13287-020-01711-w (PMC7245780; doi:10.1186/s13287-020-01711-w)
Supplement: Supplementary file 2 — Additional file 2: Table S2. Primer sequences for qRT-PCRs. [file 13287_2020_1711_MOESM2_ESM.docx]

**Additional file 2 Table S2. Primer sequences for qRT-PCRs**

| Genes | | Primer sequences (5’-3’) | | | AT (ºC) | PS (bps) | | |
| --- | --- | --- | --- | --- | --- | --- | --- | --- |
| TRF3 | | F-CATGGTTGGAAGCTGTGATG  R-AACGTTCTTTGGCACCTGTC | | 60 | 185 | |  |  |
| TBP  TBPL1 | | F-CTCACAGACTCTCACAACTGC  R-GGAGGGATACAGTGGAGTGG  F-ACAGCTACAATTTGGTCCTCAG  R-AAGTTCAGGTTCGTAACTGGC | | 60  60 | 55  234 | |  |  |
| OCT4 | | F-AGTGAGAGGCAACCTGGAGA  R-ACACTCGGACCACATCCTTC | | 60 | 81 | |  |  |
| NANOG | | F-CATGAGTGTGGATCCAGCTTG  R-CCTGAATAAGCAGATCCATGG | | 60 | 191 | |  |  |
| EOMES  T | | F-CAACATAAACGGACTCAATCCCA  R-ACCACCTCTACGAACACATTGT  F-CAGTGGCAGTCTCAGGTTAAGAAGGA  R-CGCTACTGCAGGTGTGAGCAA | | 60  60 | 54  122 | |  |  |
| MIXL1 | | F-CCGAGTCCAGGATCCAGGTA  R-CTCTGACGCCGAGACTTGG | | 60 | 58 | |  |  |
| GSC | | F-AACGCGGAGAAGTGGAACAAG  R-CTGTCCGAGTCCAAATCGC | | 60 | 89 | |  |  |
| SOX2  SOX1  SIP1  SIX1  GBX2  NEUROD1  GAPDH | | F-GGGAAATGGGAGGGGTGCAAAAGAGG  R-TTGCGTGAGTGTGGATGGGATTGTG  F-CAGTACAGCCCCATCTCCAAC  R-GCGGGCAAGTACATGCTGA  F-CGCTTGACATCACTGAAGGA  R-CTTGCCACACTCTGTGCATT  F-CTGCCGTCGTTTGGCTTTAC  R-GCTCTCGTTCTTGTGCAGGT  F-GTTCCACTGCAAAAAGTACCTCT  R-GGGACGACGATCTTAGGGTTC  F-ATGACCAAATCGTACAGCGAG  R-GTTCATGGCTTCGAGGTCGT  F-GGAGCGAGATCCCTCCAAAAT  R-GGCTGTTGTCATACTTCTCATGG | | 60  60  60  60  60  60  60 | 151  287  204  135  183  140  197 | |  |  |
|  | | | |  |  | |  |  |

F, forward; R, reverse; AT, annealing temperature; PS, product size.
