# Supplementary material for: TATA box-binding protein-related factor 3 drives the mesendoderm specification of human embryonic stem cells by globally interacting with the TATA box of key mesendodermal genes
Source: Stem Cell Res Ther. 2020 May 24;11:196. doi: 10.1186/s13287-020-01711-w (PMC7245780; doi:10.1186/s13287-020-01711-w)
Supplement: Supplementary file 3 — Additional file 3: Table S3. Primer sequences for ChIP-qPCRs. [file 13287_2020_1711_MOESM3_ESM.docx]

**Additional file 3 Table S3. Primer sequences for ChIP-qPCRs**

| Genes | | Primer sequences (5’-3’) | | | AT (ºC) | | PS (bps) | | |
| --- | --- | --- | --- | --- | --- | --- | --- | --- | --- |
| *EOMES*  *T*  *MIXL1* | | F-TTCCCTATCCGTGGCTCCTT  R-TGTACACCGCTTTTCCCTCC  F-GCTCCCGTTTTAGGAGGAAT  R-GAGGAGAGCGAAGGAGAGGT  F-GCCGTATCCTCTGCTAATGG  R-CCTAGTCCCGTCTGTCCTCA | | 60  60  60 | | 132  86  294 | |  |  |
| *GSC* | | F-GCGGCTAGGATGTTGTCGAT  R-TTTAAGGCTCTGTCCTCGGC | | 60 | | 101 | |  |  |
|  | |  |  | |  | |  |  |  |

F, forward; R, reverse; AT, annealing temperature; PS, product size.
