# Supplementary figures and images for: TATA box-binding protein-related factor 3 drives the mesendoderm specification of human embryonic stem cells by globally interacting with the TATA box of key mesendodermal genes
Source: Stem Cell Res Ther. 2020 May 24;11:196. doi: 10.1186/s13287-020-01711-w (PMC7245780; doi:10.1186/s13287-020-01711-w)

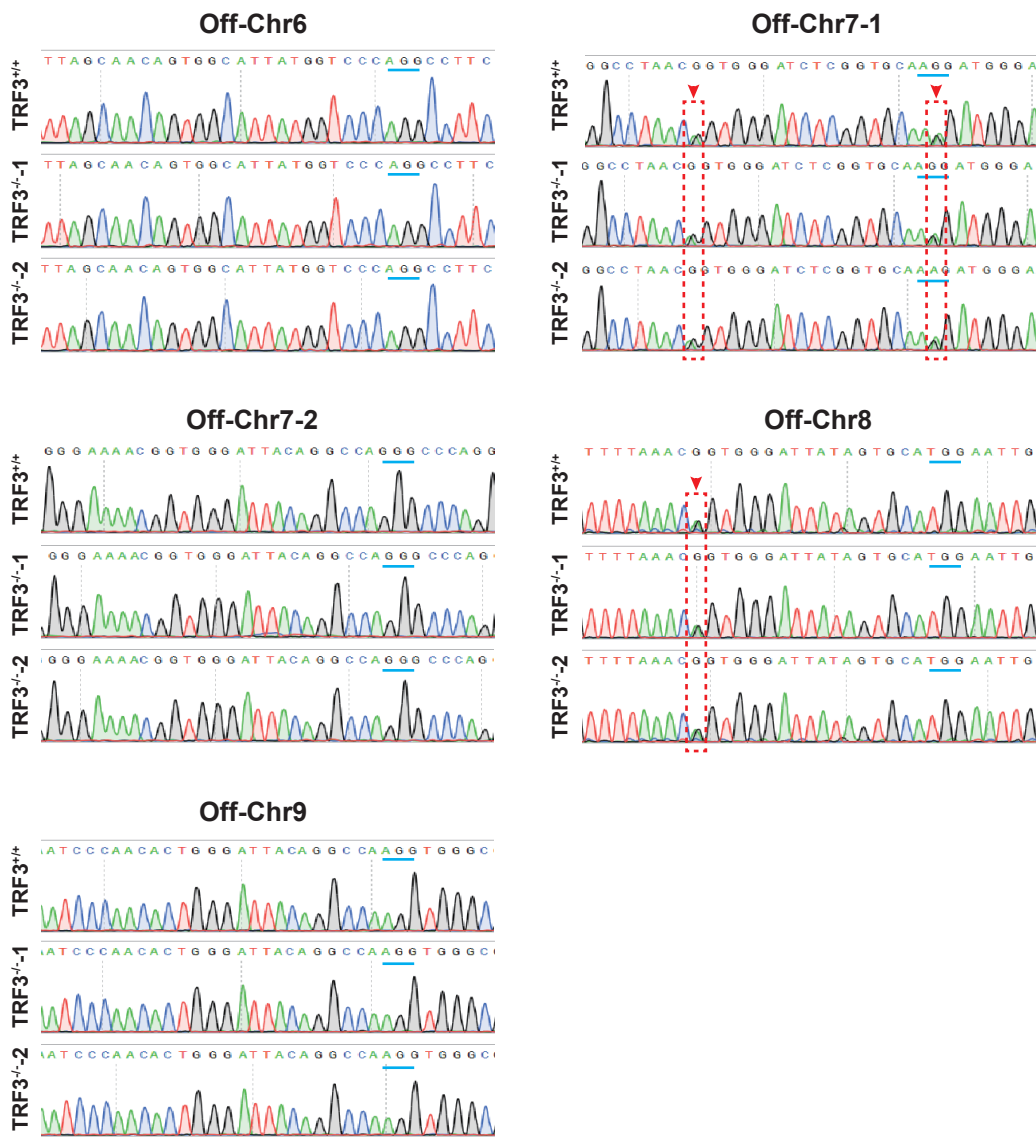

Supplement: Supplementary file 4 — Additional file 4: Figure S1. Sanger sequencing of the predicted off-target sites. The name of predicted off-target sites locates above each panel. The protospacer adjacent motif is indicated by short cyan lines. The red arrow heads and dashed rectangles indicate the single nucleotide polymorphism. [file 13287_2020_1711_MOESM4_ESM.pdf]

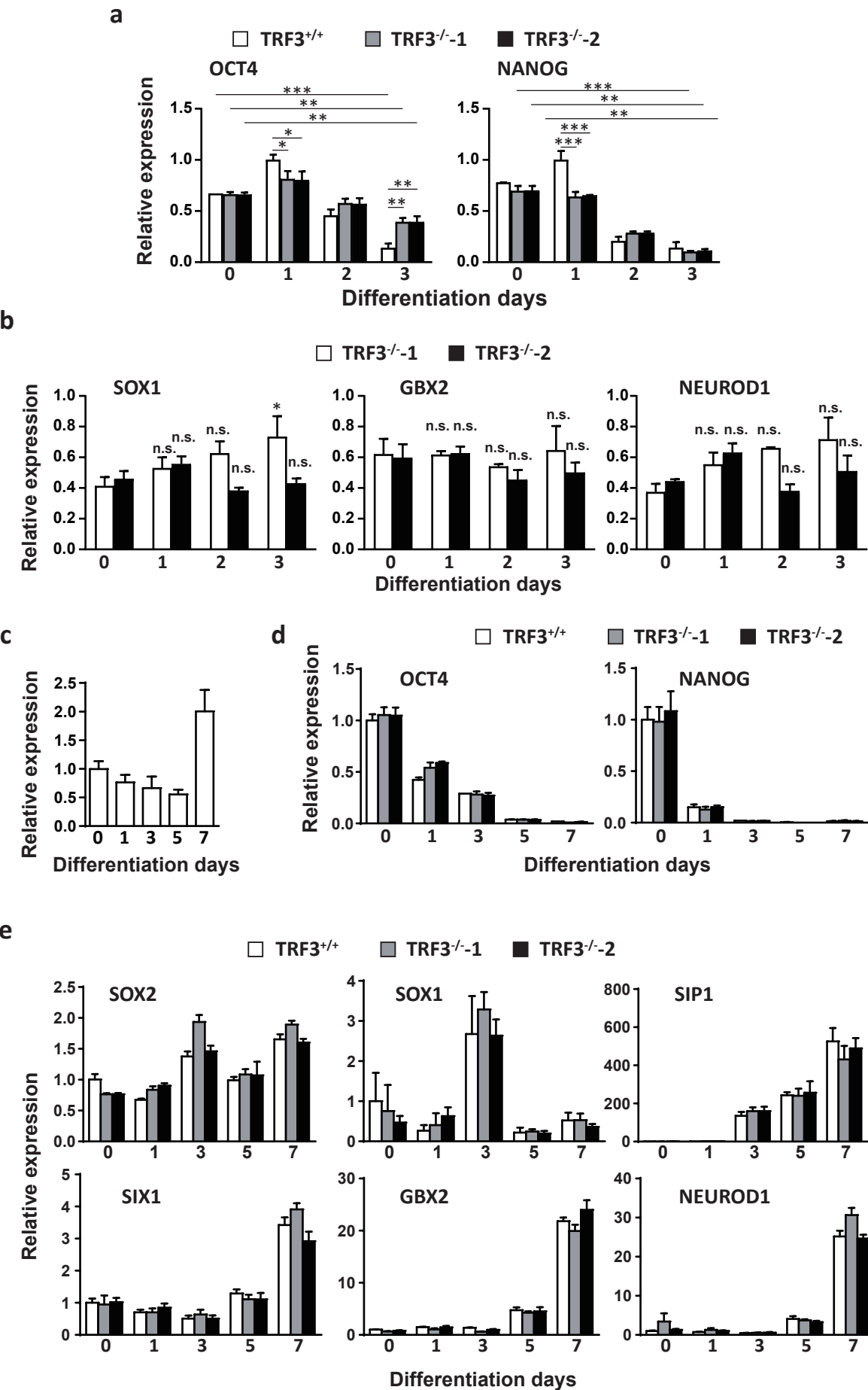

Supplement: Supplementary file 5 — Additional file 5: Figure S2. qRT-PCR analysis of pluripotency and neuroectodermal markers during the ME and neuroectodermal differentiation. (a): the expression of pluripotency markers (OCT4, NANOG) during the ME differentiation. n = 3 each. *p < 0.05, **p < 0.01, ***p < 0.001 as indicated. (b): the expression of neuroectodermal markers (SOX1, GBX2 and NEUROD1) during the ME differentiation. n = 3 each. *p < 0.05, compared with the corresponding values in undifferentiated cells. n.s., no significant difference compared with the corresponding values in undifferentiated cells. (c): qRT-PCR analysis of TRF3 during the neuroectodermal differentiation process of TRF3+/+ hESCs. (d): qRT-PCR analysis of pluripotency markers (OCT4, NANOG). n = 3 each. (e): qRT-PCR analysis of neuroectodermal markers (SOX2, SOX1, SIP1, SIX1, GBX2 and NEUROD1). n = 3 each. Data are presented as mean ± S.E.M. [file 13287_2020_1711_MOESM5_ESM.pdf]

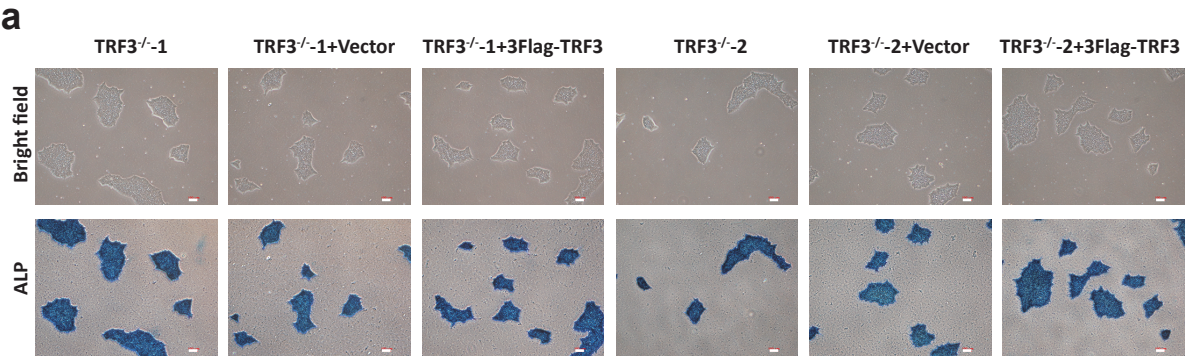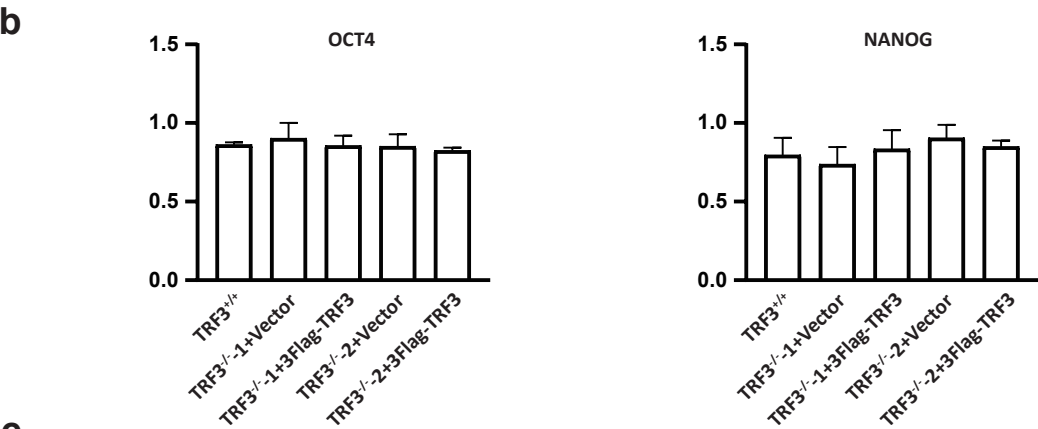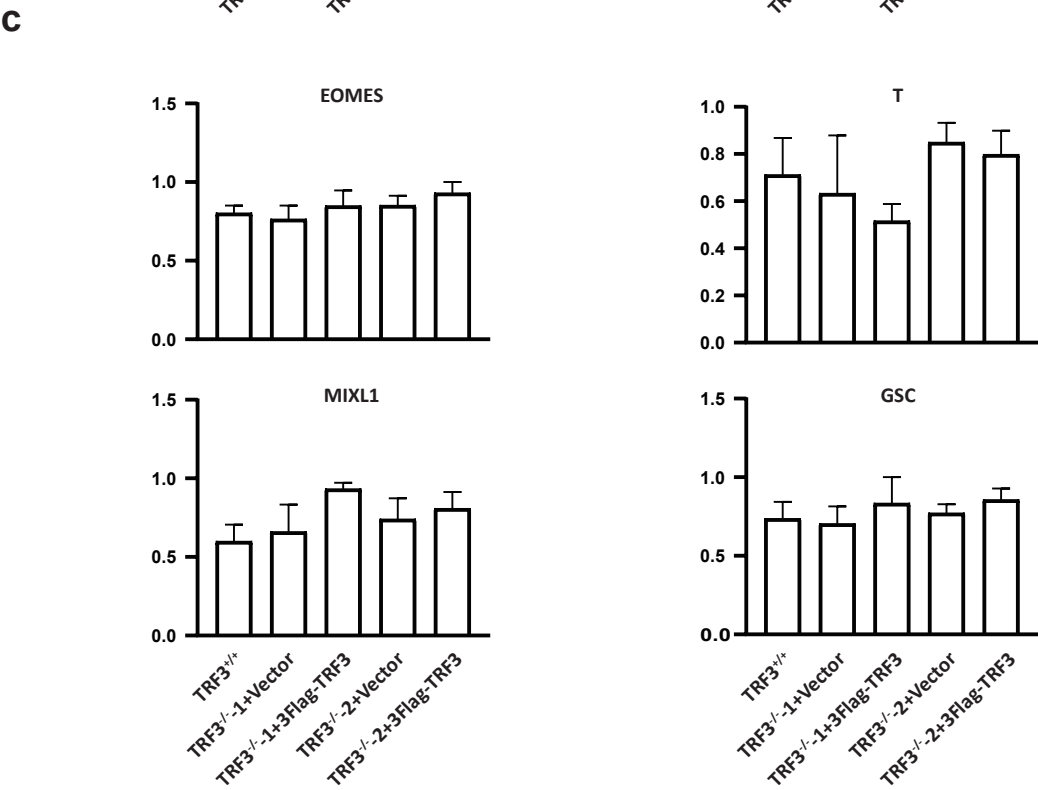

Supplement: Supplementary file 6 — Additional file 6: Figure S3. Reintroduction of TRF3 into TRF3-/--1 and TRF3-/--2 hESCs do not affect the self-renewal and the expression of ME genes in undifferentiated status. Reintroduction of TRF3 into TRF3-/-- hESCs does not affect the self-renewal and the expression of ME genes in the undifferentiated status. (a): Cell morphology and ALP activity of TRF3-/--1, TRF3-/--1+Vector, TRF3-/--1+3Flag-TRF3, TRF3-/--2, TRF3-/--2+Vector, TRF3-/--2+3Flag-TRF3 cells. Scale bar = 100 μm. (b): qRT-PCR analysis of pluripotency markers (OCT4 and NANOG) in TRF3+/+, TRF3-/--1+Vector, TRF3-/--1+3Flag-TRF3, TRF3-/--2+Vector, TRF3-/--2+3Flag-TRF3 hESCs. n = 3 each. (c): qRT-PCR analysis of ME genes (EOMES, T, MIXL1 and GSC) in TRF3+/+, TRF3-/--1+Vector, TRF3-/--1+3Flag-TRF3, TRF3-/--2+Vector, TRF3-/--2+3Flag-TRF3 hESCs. n = 3 each. [file 13287_2020_1711_MOESM6_ESM.pdf]

a

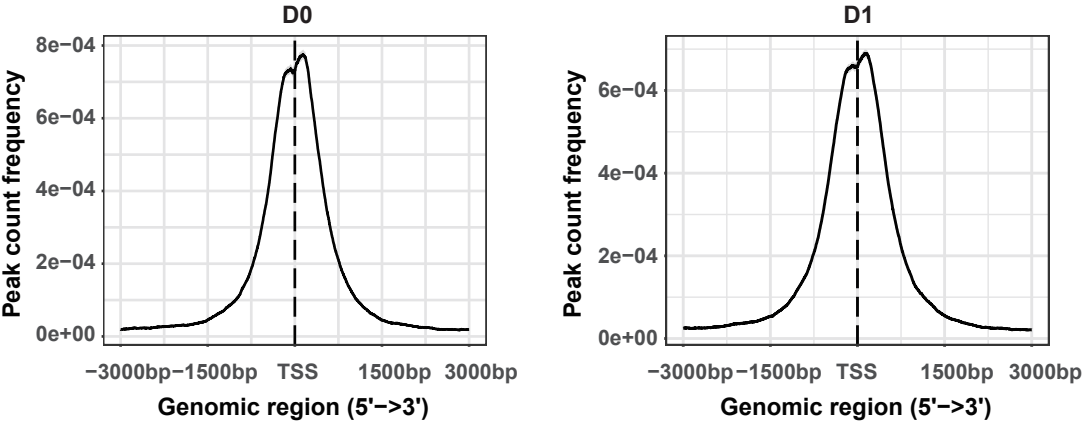

b

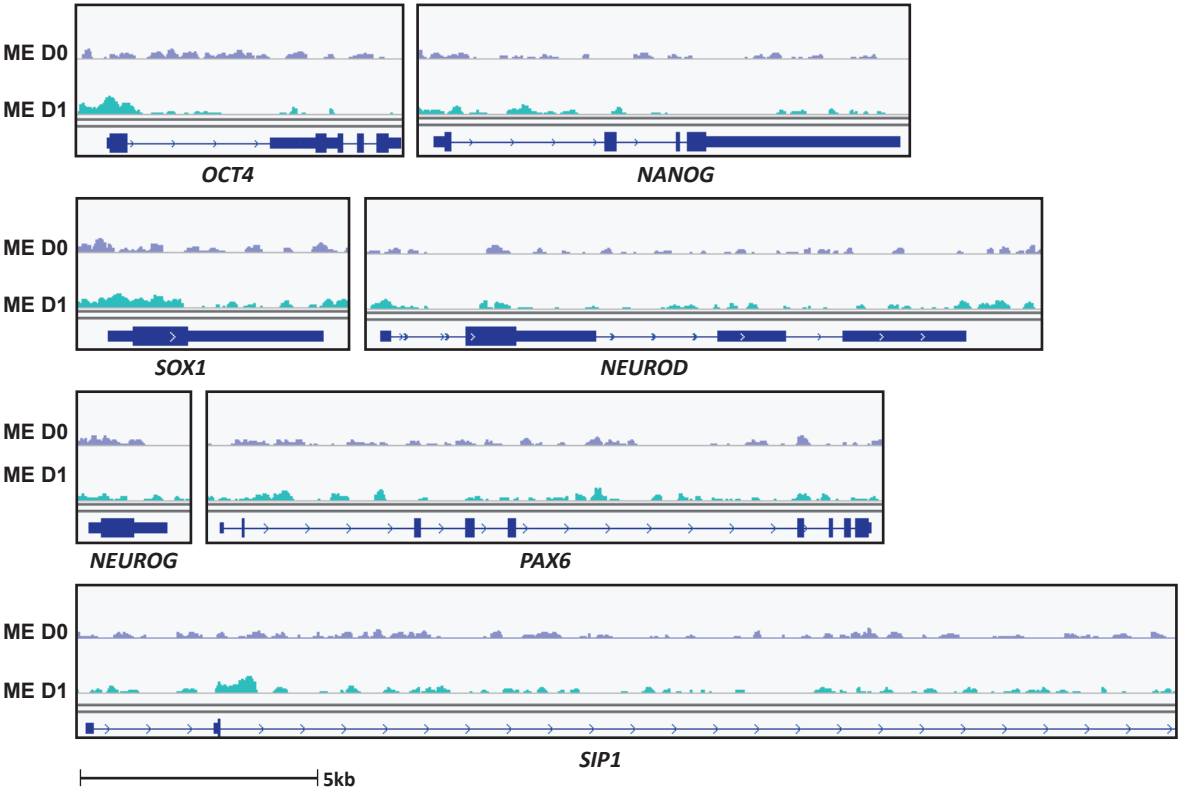

Supplement: Supplementary file 7 — Additional file 7: Figure S4. ChIP analysis of TRF3 at undifferentiated status (ME D0) and ME D1. (a): The mean peak counting frequency flanking TSS in the undifferentiated status (ME D0) and ME D1. (b): Genome browser screenshots of ChIP-seq for pluripotency markers (OCT4 and NANOG) and neuroectodermal genes (SOX1, NEUROD, NEUROG, PAX6, SIP1) in the undifferentiated status (ME D0) and ME D1. [file 13287_2020_1711_MOESM7_ESM.pdf]

a

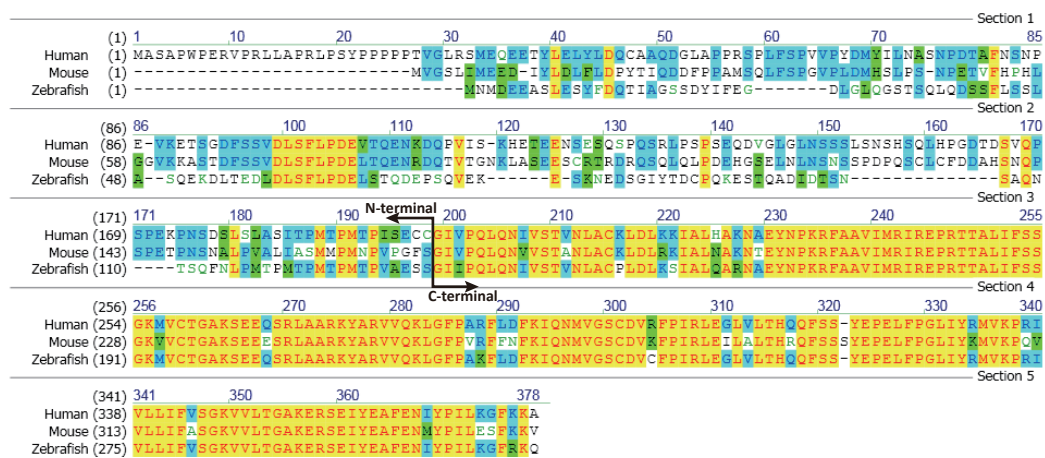

b

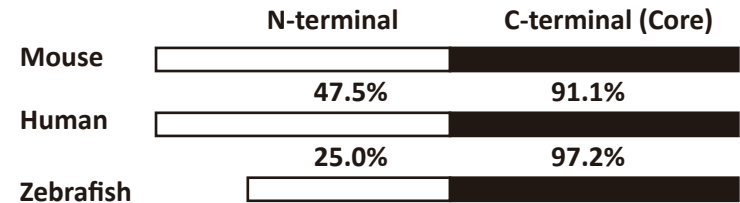

Supplement: Supplementary file 8 — Additional file 8: Figure S5. Amino acid sequence analysis of TRF3 proteins among human, mouse and zebrafish. (a): Amino acid sequence analysis of TRF3 protein. (b): Sequence conservation analysis of TRF3. [file 13287_2020_1711_MOESM8_ESM.pdf]
